# Alterations of the Gut Microbiome in Chinese Zhuang Ethnic Patients with Sepsis

**DOI:** 10.1155/2022/2808249

**Published:** 2022-05-20

**Authors:** Jieyang Yu, Hongping Li, Jingjie Zhao, Yanhua Huang, Chunlei Liu, Pengfei Yang, Dinggui Lu, Jian Song, Lingzhang Meng

**Affiliations:** ^1^Center for Systemic Inflammation Research (CSIR), School of Preclinical Medicine, Youjiang Medical University for Nationalities, Baise, Guangxi Province, China; ^2^Baise Maternal and Child Hospital, Baise, Guangxi Province, China; ^3^Neonatal Intensive Care Unit, Shenzhen Children's Hospital, Shenzhen, Guangdong Province, China; ^4^Life Science and Clinical Research Center, The Affiliated Hospital of Youjiang Medical University for Nationalities, Baise, Guangxi Province, China; ^5^Department of Critical Care Medicine, People's Hospital of Baise, Baise, Guangxi Province, China; ^6^Trauma Center, Affiliated Hospital of Youjiang Medical University for Nationalities, Baise, Guangxi Province, China

## Abstract

**Objectives:**

Sepsis is characterized as a dysregulated host immune response to infection and has been known to be closely associated with the gut microbiome. This study was aimed at investigating the gut microbial profiles of Zhuang ethnic patients with sepsis.

**Method:**

Eleven Zhuang ethnic patients with sepsis and 20 healthy individuals (controls) were recruited at the Baise City People's Hospital, China. Their gut microbial community profiles were analyzed by 16S rRNA gene sequencing using the Illumina MiSeq system.

**Results:**

The gut microbial community of patients with sepsis was significantly altered compared to that of the healthy individuals based on the results of principal coordinate analysis and microbial ecological networks. Additionally, significantly lower microbial alpha diversity was observed in patients with sepsis than in healthy individuals. In particular, the enrichment of *Bilophila*, *Burkholderia*, *Corynebacterium*, and *Porphyromonas*, along with the reduced abundance of a large number of short-chain fatty acid-producing microbes, including *Roseburia*, *Bifidobacterium*, *Faecalibacterium*, *Coprococcus*, *Blautia*, *Clostridium*, *Ruminococcus*, and *Anaerostipe* was observed in patients with sepsis compared to the control group. Moreover, patients with sepsis could be effectively classified based on the abundance of these bacteria using a support vector machine algorithm.

**Conclusion:**

This study demonstrated significant differences in the gut microbiome between Zhuang ethnic patients with sepsis and healthy individuals. In the future, it is necessary to determine whether such alterations are the cause or consequence of sepsis.

## 1. Introduction

Sepsis is a life-threatening organ dysfunction syndrome characterized as a dysregulated immune response to infection [1, 2]. It is a major public health problem, as it accounts for a remarkable proportion of deaths of hospitalized patients worldwide, especially those in the intensive care unit [2–4]. Therefore, the pathogenesis of sepsis has attracted wide attention in recent years. Most of the studies aiming to unravel the etiology of sepsis have focused on the role of the host immune responses [5]. Culture-independent methods such as 16S rRNA and shotgun metagenomic sequencing have revealed that the gut microbiome is crucial for the development and maturation of the host immune system [6]. Some microbes directly regulate host immune homeostasis by enhancing immunoglobulin A production and T-helper-17 cell differentiation [7]. Additionally, some of their products, such as lipopolysaccharides and short-chain fatty acids (SCFAs), stimulate Toll-like receptor 4- (TLR-4-) positive epithelial cells and dendritic cells [8] and activate the development of regulatory T cells [7].

Increasing evidence supports the existence of a link between the gut microbiome and sepsis [9, 10]. Several studies using preclinical models and on hospitalized patients have reported that the risk of bloodstream infections and critical illness increases when gut microbiome homeostasis is disrupted [11–13]. Zeng et al. found that some gut bacteria can induce the production of protective IgG through the secretion of specific antigens, thereby contributing to the control of systemic infections [14]. Deshmukh et al. demonstrated that the gut microbiome regulates sepsis in neonatal mice by affecting neutrophil homeostasis [15]. Additionally, probiotic supplementation is associated with a reduced risk of sepsis in patients undergoing elective gastrointestinal surgery [16] and also reduces the risk of late-onset sepsis in preterm infants [17]. Finally, it has been reported that fecal microbiota transplantation can restore the innate immune response of patients with sepsis, contribute to pathogen clearance, and regulate SCFA-producing microbes [18, 19].

Altogether, these findings support the association between gut microbiota disruption and the risk of sepsis. This study was aimed at further studying this relationship in a cohort of patients belonging to the Zhuang ethnicity. For this purpose, we investigated the characteristics of the gut microbiota in patients with sepsis using 16S rRNA gene sequencing.

## 2. Methods

### 2.1. Study Cohort

All individuals in this study were recruited from Baise City People's Hospital (Guangxi Zhuang Autonomous Region, China) from January to April 2021. All of them provided written informed consent. After excluding the patients who had undergone organ transplantation, long-term immunosuppressive therapy, or developed tumors, a total of 31 individuals were recruited for the analysis. In total, 11 patients were categorized into the sepsis (SEP) group, while 20 healthy individuals who received no antibiotics or probiotics in the last 3 months were categorized as the healthy control (CTRL) group. Sepsis was diagnosed according to the Sepsis 3.0 guidelines (https://rebelem.com/sepsis-3-0). Clinical information was collected and summarized by well-trained clinicians according to standard procedures. The study protocol was approved by the Ethical Committee of Baise City People's Hospital.

### 2.2. Stool Sample Collection and Sequencing

Stool samples were collected in sterile containers, snap-frozen in dry ice, and stored in the research laboratory at −80°C until further use. The samples of patients with sepsis were collected prior to antibiotic treatment. Total bacterial DNA was extracted from stool samples using the QIAamp Fast DNA Stool Mini Kit (Qiagen, Germany) following the manufacturer's instructions. The V3-V4 region of the 16S rRNA gene was amplified from the extracted DNA using PCR primers 338F (5′-ACTCCTACGGGAGGCAGCAG-3′) and 806R (5′-GGACTACHVGGGTWTCTAAT-3′). PCR products were mixed at equidensity ratios. Qiagen Gel Extraction Kit was used for purification (Qiagen, Germany). The prepared library was sequenced on an Illumina MiSeq platform with a 300 bp paired-end read mode.

### 2.3. Gut Microbiota and Statistical Analyses

16S rRNA gene sequencing analysis, including raw sequence filtering and taxonomic classification, was performed as previously described [20]. The bioinformatic software package QIIME2 (version 2020.11) was used to analyze the 16S rRNA gene sequences [21]. The paired reads were assembled and denoised with the DADA2 package [22] using the “qiime dada2 denoise-paired” command in QIIME2. The command “qiime feature-classifier classify-sklearn” was used to assign sequences to taxonomy against the Greengenes database. Metrics of Shannon's index, Pielou's evenness index, observed feature number, and unweighted and weighted UniFrac distances were calculated using the command “qiime phylogeny align-to-tree-mafft-fasttree” and “qiime diversity core-metrics-phylogenetic” at a sampling depth of 10,000 reads. Metabolic function prediction was performed using PICRUSt2 software [23] with the “qiime picrust2 full-pipeline” command.

Principal coordinate analysis (PCoA) based on unweighted and weighted UniFrac distances was used to estimate differences in the beta diversity, accompanied by permutational multivariate analysis of variance (PERMANOVA) to assess the significance of community dissimilarity using the “vegan” R package. Linear discriminant analysis (LDA) was used to estimate the differentially abundant taxa with the linear discriminate analysis of effect size (LEfSe) software between the two groups (*P* < 0.05) [24]. The microbial ecological network was constructed using SpiecEasi software [25] and visualized using Gephi software. STAMP software was used to investigate the significantly different metabolic pathways between the two groups [26].

Characteristics were summarized as means (± standard deviations) for continuous variables and ratio (%) for categorical variables. Differences in these characteristics were assessed using *t*-tests and *X*^2^ tests. All analyses were performed using R software (v 3.6.3). The differences were considered statistically significant at *P* < 0.05.

A classification model was built to classify patients with sepsis based on the significantly different genera using a support vector machine (SVM) algorithm with 10-fold cross-validation. The area under the curve (AUC) was calculated to evaluate the model's performance.

## 3. Results

### 3.1. Characteristics of Study Participants

A total of 31 Zhuang ethnic individuals were enrolled in this study. All participants were aged between 65 and 98 years. The sociodemographic and clinical characteristics of all the participants are summarized in Table 1. According to the statistical analysis results, there were no significant differences in age or sex between the groups. Compared to the CTRL group, patients with sepsis had higher levels of C-reactive protein (CRP), interleukin- (IL-) 6, and heparin-binding protein (HBP).

### 3.2. Overall Structure Diversity of Gut Bacterial Communities

**Figure 1:**
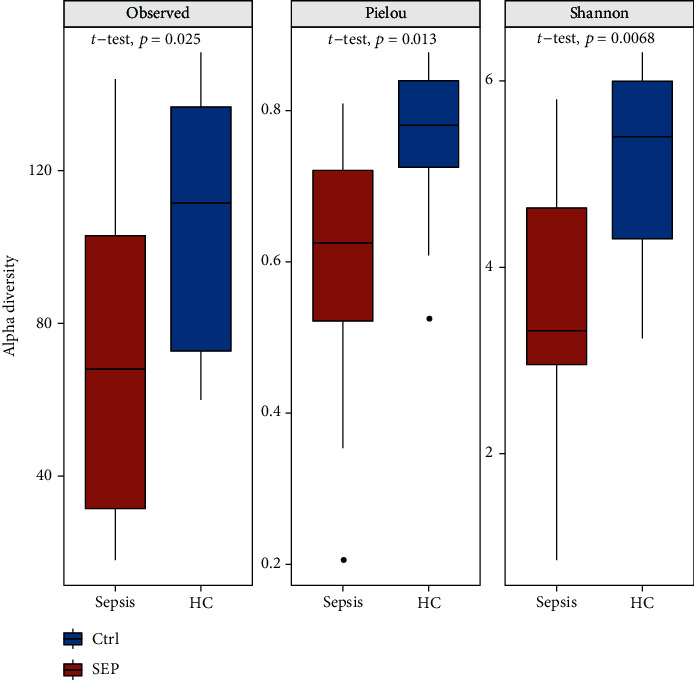
Comparisons of alpha diversity indexes between sepsis (SEP) and healthy control (Ctrl) group. Significant lower observed feature number, Pielou, and Shannon indexes were observed in patients with sepsis.

To evaluate the overall characteristics of the gut microbiome, alpha diversity estimators such as observed feature number, Pielou, and Shannon indexes were compared. As shown in Figure 1, the observed feature number of the CTRL group was 105.5 ± 33.57, which was significantly higher than that of the SEP group (68.36 ± 43.49, *P* < 0.05). The Pielou index was also significantly lower in the SEP group (0.59 ± 0.19) than in the CTRL group (0.77 ± 0.09, *P* < 0.05). Moreover, patients with sepsis had a significantly lower value of the Shannon index than that of the healthy individuals (3.48 ± 1.56 versus 5.08 ± 0.96, *P* < 0.05).

**Figure 2:**
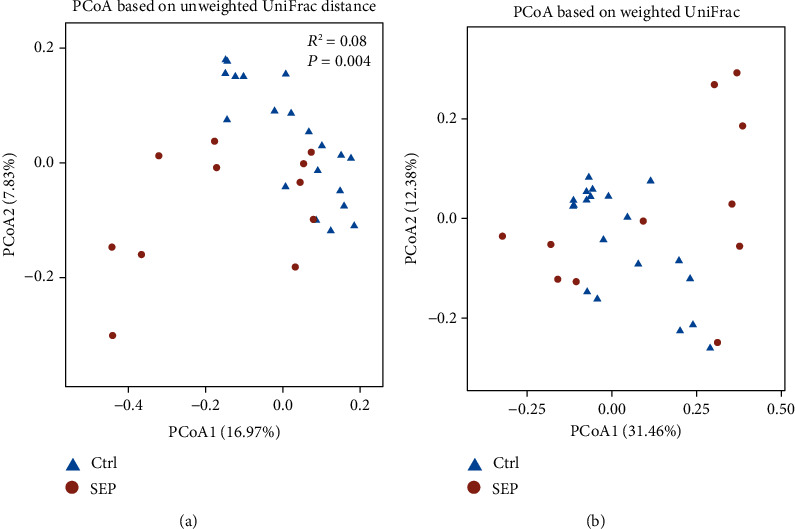
Principal coordinate analysis (PCoA) plot based on unweighted and weighted UniFrac distances with PERMANOVA analysis. (a). The PCoA plot based on unweighted UniFrac distance. (b). The PCoA plot based on weighted UniFrac distance.

PCoA was performed on weighted and unweighted UniFrac distances using PERMANOVA. Obvious separations could be observed between SEP and healthy groups both on unweighted (*R*^2^ = 0.08, *P* = 0.004, Figure 2(a)) and weighted UniFrac distances (*R*^2^ = 0.10, *P* = 0.007, Figure 2(b)).

### 3.3. Bacterium Analysis of SEP and CTRL Groups

**Figure 3:**
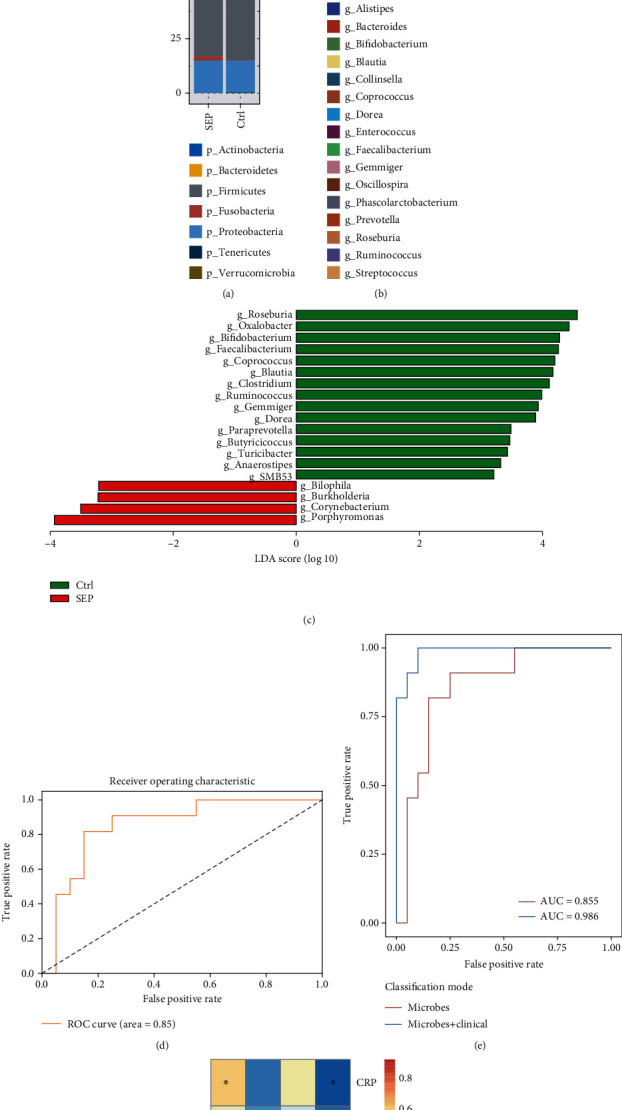
Microbial profiles of the gut microbiota in the sepsis (SEP) and healthy control (Ctrl) groups. (a). Relative abundances of the dominant phyla. (b). Relative abundances of the abundant genera. (c). Differences in microbial genera between SEP and Ctrl groups. (d). Receiver operating characteristic curve of the classification mode for sepsis based on different genera using support vector machine algorithm. (e). Receiver operating characteristic curve of the classification mode for sepsis based on different genera in combination of clinical features using support vector machine algorithm. (f). Correlations between some abundant microbes with C-reactive protein (CRP), heparin-binding protein (HBP), and (CRP) and interleukin-6 (IL-6) levels separately.

Gut microbiome communities were dominated by the phyla Actinobacteria, Bacteroidetes, Firmicutes, Fusobacteria, Proteobacteria, Tenericutes, and Verrucomicrobia in both groups (Figure 3(a)). The most abundant genera in both groups were *Alistipes*, *Bacteroides*, *Bifidobacterium*, *Blautia*, *Collinsella*, *Coprococcus*, *Dorea*, *Enterococcus*, *Faecalibacterium*, *Gemmiger*, *Oscillospira*, *Phascolarctobacterium*, *Prevotella*, *Roseburia*, *Ruminococcus*, and *Streptococcus* (Figure 3(b)). In the SEP group, the mean relative abundances of *Bifidobacterium* (0.08% versus 3.69%)*, Blautia* (3.24% versus 6.91%)*, Coprococcus* (1.59% versus 4.98%)*, Faecalibacterium* (1.10% versus 4.48%)*, Gemmiger* (0.64% versus 2.32%)*, Roseburia* (0.25% versus 7.59%)*, Ruminococcus* (5.37% versus 11.03%), and *Streptococcus* (2.42% versus 3.53%) were decreased compared to the CTRL group. The mean relative abundances of *Bacteroides* (10.23% versus 6.05%), *Enterococcus* (27.9% versus 0.09%), and *Prevotella* (5.53% versus 2.95%) were comparatively increased in the SEP group.

Furthermore, we used LEfSe software to investigate the significant differences in genera between the individuals with and without sepsis. The results demonstrated that the relative abundance of *Bilophila*, *Burkholderia*, *Corynebacterium*, and *Porphyromonas* was higher in the SEP group than in the CTRL group, whereas that of *Roseburia, Oxalobacter, Bifidobacterium, Faecalibacterium, Coprococcus, Blautia, Clostridium, Ruminococcus, Gemmiger, Dorea, Paraprevotella, Butyricicoccus, Turicibacter, Anaerostipes*, and *SMB53* was lower in the SEP group than in the CTRL group (Figure 3(c)).

Next, to explore the efficacy of classification based on these significantly different genera in the SEP group, we built a classification model using an SVM algorithm with 10-fold cross-validation. The receiver operating characteristic curve of the model is shown in Figure 3(d) and has an AUC of 0.85. After combining with the CRP, IL-6, and HBP levels, the AUC increased to 0.986 (Figure 3(e)).

Additionally, Pearson correlation analysis was performed to evaluate the associations between the differential genera and CRP, HBP, and IL6 levels (Figure 3(f)). The abundance of the genus *Bilophila* was positively correlated with CRP (*R*^2^ = 0.55, *P* < 0.01) and HBP (*R*^2^ = 0.36, *P* = 0.045) levels; that of *Porphyromonas* was positively correlated with IL-6 level (*R*^2^ = 0.94, *P* < 0.01); that of *Bacteroides* was positively correlated with CRP level (*R*^2^ = 0.40, *P* = 0.026); and that of *Prevotella* was positively correlated with IL-6 level (*R*^2^ = 0.85, *P* < 0.01).

### 3.4. Functional Alterations between SEP and CTRL Groups

**Figure 4:**
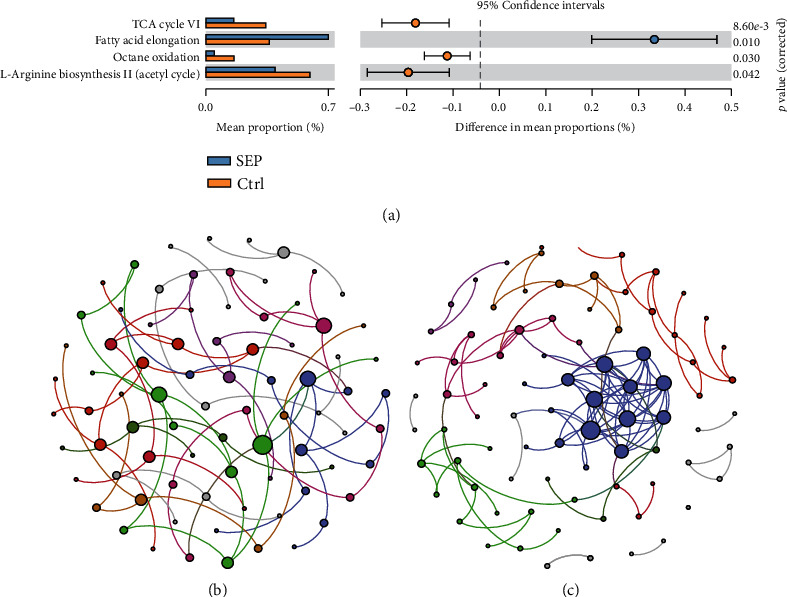
Functional metabolic pathways and microbial ecological network in the SEP and Ctrl groups. (a). Significantly different metabolic pathways between SEP and Ctrl groups. (b). Microbial ecological network of the gut microbiome in the patients with sepsis. (c). Microbial ecological network of the gut microbiome in the healthy individuals.

We analyzed the metabolic pathways in the individuals belonging to both groups. The predicted functions were performed using PICRUSt2 software. The fatty acid elongation pathway was significantly enriched in the gut microbiome of the SEP group compared to that of CTRL. The tricarboxylic acid (TCA) cycle VI, octane oxidation, and L-arginine biosynthesis II (acetyl cycle) were higher in the gut microbiome of the CTRL group (Figure 4(a)).

### 3.5. Different Microbial Ecological Networks between SEP and CTRL Groups

Microorganisms in the gut construct a stable ecological network with cooccurrence, competition, and antagonistic relationships. To investigate the microbial network at the genus level, we used the SpiecEasi software. The microbial ecological network of the CTRL group presented 84 nodes and 131 (Figure 4(b)). However, the microbial ecological network of patients with sepsis was simpler, presenting only 80 nodes and 77 edges (Figure 4(c)).

## 4. Discussion

Sepsis is a major public health issue, with more than one million sepsis-related deaths occurring in China in 2015 (Li et al., 2018). Increasing evidence supports that gut microbial disruption predisposes patients to sepsis, thereby presenting as a potential therapeutic target in sepsis management [2, 10]. As the Zhuang ethnic minority is the largest Chinese minority group, in this study, we aimed to characterize the gut microbiota in patients belonging to the Zhuang ethnicity suffering from sepsis. Using PCoA, we demonstrated that patients with sepsis present a significantly different microbial community and a different microbial ecological network than the healthy individuals. In addition, patients with sepsis had significantly lower microbiome alpha diversity, which is consistent with a previous study [27]. Additionally, a previous study reported that enhancing gut microbiome diversity in mice could increase sepsis survival by regulating the immune response [28]. Therefore, increasing the gut microbiome diversity may be beneficial in the treatment of patients with sepsis.

Increases in pathogenic intestinal bacteria and exuberant immune responses are often observed in patients with sepsis [10]. In this study, we observed that the commonly hospital-acquired pathogen *Enterococcus* was relatively frequent in patients with sepsis, which was in accordance with the results of a previous study [29]. Furthermore, compared to the healthy individuals, there were four genera significantly enriched in patients with sepsis, and 15 genera significantly decreased. Based on the abundance of these microbes, patients could be effectively classified as healthy individuals or patients with sepsis using an SVM algorithm. Among the four increased genera, *Bilophila* can produce lipopolysaccharides to stimulate the immune system via TLR-4 [30]. *Porphyromonas gingivalis* can regulate host innate immune signaling and induce inflammation [31]. In this study, the relative abundance of *Bilophila* was positively correlated with CRP and HBP levels, and the relative abundance of *Porphyromonas* was positively correlated with the IL-6 level. Their relative increase in patients with sepsis might prime the immune system for a robust proinflammatory response. Additionally, among the significantly decreased genera, *Roseburia, Bifidobacterium, Faecalibacterium, Coprococcus, Blautia, Clostridium, Ruminococcus,* and *Anaerostipes* can produce SCFAs [32]. Decreased production of SCFAs can enhance intestinal epithelial cell function [33], activate the development of regulatory T (Treg) cells [34], and decrease nuclear factor kappa B- (NF-*κ*B-) regulated proinflammatory cytokines [35]. Hence, the significant decrease in SCFA-producing bacteria in patients with sepsis observed in this study might have adverse consequences for both gut integrity and systemic immunity.

Since an individual is born, the gut microbiota and host live in symbiotic homeostasis that influences the immune function, including T cell differentiation and activation, cytokine production, and local barrier function [2]. The alterations of the gut microbiome observed in this study might predispose individuals to sepsis by favoring the proliferation of pathogenic bacteria and decreasing the production of SCFAs, which would promote a dysregulated immune response. However, the findings of this study should be considered in light of its limitations, and larger prospective studies should be conducted to confirm this hypothesis. In this study, the sample size was relatively small, and all the individuals in this study were recruited from a single geographic area without consideration of different demographics. Additionally, the alterations in microbial function and whether such microbial alterations in this study were the causes or consequences of sepsis were not clear. Studies with larger sample sizes, including individuals from different regions, conducting metagenomic and metaproteomic analyses, and experimental studies should be conducted in the future to further answer these questions.

Limitations of this study include, for example, lack of the delineation and the intermodulation between specific bacterium and host immune system; the relationship between gut microbiota and prognosis has not been explored due to limited sample size and the short-term observation period; in addition, this study failed to collect the biopsies from pregnant women which could be important for newborn cases. The above limitations deserve to draw attentions and should be answered in future study.

In conclusion, we identified dysbiosis of the gut microbiome in Zhuang ethnic patients with sepsis. Disruption of the gut microbiome may serve as a potential biomarker for early detection of sepsis risk. Our findings could help improve the understanding of the role of the gut microbiome in sepsis etiology and support preventive strategies based on the modulation of the gut microbiome.

## Figures and Tables

**Table 1:** Demographic and clinical characteristics of the two groups.

Characteristic	SEP (*n* = 11)	Ctrl (*n* = 20)	*P* value
Age (years)	74.45 ± 10.23	70.35 ± 1.57	0.09
Male/female	7/4	12/8	1
CRP (mg/L)	48.26 ± 61.83	1.98 ± 2.78	<0.01^∗∗^
IL-6 (pg/mL)	102.18 ± 157.66	27.39 ± 33.42	<0.05^∗^
HBP (ng/mL)	66.17 ± 63.82	8.86 ± 4.36	<0.01^∗∗^

SEP: Sepsis; Ctrl: Healthy controls; CRP: C-reactive protein; IL-6: Interleukin-6; HBP: Heparin-binding protein.

## Data Availability

The datasets and code generated and analyzed in this study are available from the corresponding author on request.
